# Strategies to strengthen COVID-19 vaccine uptake and improve vaccine equity in U.S. Territories and Freely Associated States during the first six months of vaccine rollout

**DOI:** 10.1016/j.vaccine.2024.05.005

**Published:** 2024-05-07

**Authors:** Ashley Tippins, Jeniffer Concepción Acevedo, Francisco S. Palomeque, Kelsey C. Coy, Phillippa Chadd, Daniel Stowell, Oluwatomiloba Ademokun, Carter Apaisam, Merlyn Basilius, Richard Brostrom, Ivan O. Garcia Collazo, Justa Encarnacion, Iris Cardona Gerena, Thane Hancock, Tai Hunte-Ceasar, Peter Judicpa, Michele Leon-Guerrero, Milton Martinez, Yolanda Masunu, Heather Pangelinan, Emman Parian, Daisy Pedro

**Affiliations:** a Centers for Disease Control and Prevention, USA; b Eagle Health Analytics, USA; c Federated States of Micronesia Ministry of Health, USA; d Palau Ministry of Health & Human Services, USA; e Puerto Rico Department of Health, USA; f US Virgin Islands Department of Health, USA; g Guam Department of Health & Social Services, USA; h American Samoa Department of Health, USA; i Commonwealth Healthcare Corporation, USA; j Republic of the Marshall Islands Ministry of Health, USA

**Keywords:** COVID-19 vaccine, U.S. territories and freely associated states, Vaccine distribution, Vaccine equity

## Abstract

The eight U.S. territories and freely associated states (TFAS) have historically faced unique social and structural barriers in the implementation of vaccination programs due to geographic remoteness, a high prevalence of socioeconomic disparities, increasing prevalence of natural disasters, limited vaccine providers and clinics, difficulties with procurement and shipping, and difficulty tracking highly mobile populations. In the months leading up to emergency authorizations for the use of COVID-19 vaccines, the TFAS developed tailored vaccination strategies to ensure that key at-risk populations received timely vaccination, and successfully implemented these strategies during the first six months of the vaccine rollout. Subject matter experts supporting the Centers for Disease Control and Prevention’s COVID-19 Response recognized the unique historical, geographic, social, and cultural dynamics for residents in the TFAS and worked with partners to prevent, detect, and respond to the pandemic in these jurisdictions. As a result of innovative partnerships and vaccine distribution strategies, vaccine equity was improved in the TFAS during the COVID-19 vaccine rollout.

## 1. Introduction

COVID-19 has had a significant impact on the world and has exacerbated existing health inequities [1,2]. Vaccination has become one of the key strategies to mitigate the spread of the SARS-CoV-2 virus. The 64 state, territorial, and local immunization programs who receive federally funded vaccines from the United States (U.S.) Centers for Disease Control and Prevention’s (CDC) domestic immunization program developed phased COVID-19 vaccine distribution strategies and rapidly implemented their vaccine plans beginning on December 14, 2020 [3].

Within the U.S. domestic immunization program, there are five participating U.S. territories and three freely associated states (TFAS). These include six U.S.-affiliated Pacific Island (USAPI) jurisdictions and two U.S. territories in the Caribbean: American Samoa, Guam, the Commonwealth of the Northern Mariana Islands (CNMI), the Federated States of Micronesia (FSM), the Republic of Palau, the Republic of the Marshall Islands (RMI), Puerto Rico (PR), and the U.S. Virgin Islands (USVI). The three freely associated states (FSM, Palau, and RMI) are independent nations that have a special association with the United States which enables them access to U.S. domestic health programs [4]. While the TFAS share similarities to one another related to their association with the United States and to some extent their geographies, they are distinct culturally, economically, and in size and geographic distribution of their populations. Population sizes in the TFAS range from 17,614 in Palau to 3.3 million in Puerto Rico (Supplementary Table 1). In FSM and RMI, approximately one-third of the population live on remote outer islands.

The TFAS have historically faced unique social and structural barriers in the implementation of public health programs. Immunization program staff report that the geographic remoteness – particularly in jurisdictions with far-reaching outer islands – impacts vaccine distribution and delivery, strains limited resources, and adversely affects vaccination coverage. Populations living in the more remote or difficult-to-access regions of the jurisdictions have historically had disproportionately low vaccination coverage, among other health inequities [5]. Additional challenges to maintaining adequate vaccination coverage in the past have included a high prevalence of socioeconomic disparities, limited healthcare providers and clinics with vaccine storage and handling capacity, difficulties with procurement and shipping, difficulty tracking highly mobile populations, and increasing prevalence of natural disasters [6]. Pre-pandemic vaccination data show starkly lower coverage in the TFAS for routine childhood vaccines and adult vaccines such as seasonal influenza, compared to the U.S. states [5,7,8]. For example, cumulative coverage for seasonal influenza vaccine in the 2019–2020 season was 42.1% for the U.S. overall. However, seasonal influenza vaccine coverages for all TFAS were lower, with the highest coverage reaching 29.6% in CNMI, and these trends had been similar across years prior to the pandemic (Supplementary Table 2).

The COVID-19 pandemic impacted all eight TFAS, however the extent, the timeline, and the public health response efforts varied between them. Early in the pandemic, TFAS populations living in the United States mainland were found to be disproportionately affected by COVID-19. For example, in March-June 2020, an evaluation of the impact of COVID-19 on the Marshallese population living in Arkansas found that this group had the highest COVID-19 case rate of any ethnicity, as well as the highest recorded case fatality proportion [9]. It was predicted that once COVID-19 reached the Marshall Islands and the other under-resourced TFAS jurisdictions they could experience even greater mortality due to limited resources and health system capacity in these jurisdictions. Considering this, RMI and all the TFAS implemented various mitigation strategies to limit the spread of disease in the months before vaccine was available.

Puerto Rico was one of the first TFAS to impose a strict curfew and closure of non-essential businesses on March 15, 2020 [10]. The USVI closed schools on March 16, 2020 and the following week issued stay at home orders and closures of non-essential businesses [11]. The USAPI jurisdictions initially implemented varying combinations of mitigation measures, using a combination of strict border controls, pre-departure and arrival quarantine and testing requirements, or arrival-only quarantine and testing systems to protect their health systems and populations while treatments and prevention strategies were developed, substantially delaying introduction of the virus in their jurisdictions [12]. The TFAS were also some of the last jurisdictions to lift policies introduced for mandatory face masking and crowd management. Other mitigation strategies like community-based test-to-treat programs mitigated access barriers and improved equitable access to COVID-19 oral antiviral therapeutics. In the Pacific TFAS, COVID-19 antiviral therapeutics were available beginning in October 2021, including Paxlovid after its emergency use authorization in December 2021. The rapid implementation of community test-to-treat centers allowed individuals to be tested using rapid antigen tests and, for persons with risk factors for severe COVID-19 disease who tested positive, therapeutics were provided on-site. A study in Palau that found no deaths for any of the COVID-19 patients who received novel therapeutics suggested that therapy at time of diagnosis provided additional protection against severe disease [13].

In the months leading up to emergency authorizations for the use of COVID-19 vaccines, all 64 immunization programs developed comprehensive vaccine implementation plans. Due to their unique populations and needs, the TFAS developed tailored vaccination strategies to ensure that key at-risk populations received timely vaccination.

CDC worked with other federal agencies, local partners, and the TFAS immunization programs to overcome social and structural barriers and help implement systems to prevent, detect, and respond to the pandemic in these jurisdictions. This report aims to describe the innovative partnerships and vaccine distribution strategies that contributed to achieving high vaccine uptake and improving vaccine equity (by reducing geographic disparities in access to vaccine) in the TFAS during the initial six months of COVID-19 vaccine rollout.

## 2. Methods

To describe partnership development and vaccine distribution strategies used in the TFAS during the initial COVID-19 vaccine rollout, qualitative data was collected from immunization program staff and CDC subject matter experts who supported the jurisdictions during the COVID-19 response. Data from field notes, coordination call notes, and responses to informal key informant interviews were collected by the authors and combined with desk review of supporting information from published news articles, press releases, and other publications. Data from all sources were compiled and coded to identify themes under two key content areas: approaches to vaccination distribution/logistics and collaborations/partnerships.

Quantitative vaccination data were collected through accessing publicly available vaccine distribution and vaccination coverage data from the CDC COVID Data Tracker website, collecting internal coverage data tables provided directly by jurisdictional immunization program staff (for USVI and PR), and by analyzing data from jurisdictional immunization information systems (IIS) (for USAPI). Data from IIS were analyzed using SAS v. 9.4.

### 2.1. Vaccine distribution strategies

Emergency use authorization (EUA) for the Pfizer-BioNTech COVID-19 vaccine was granted by the Food and Drug Administration (FDA) on December 10, 2020, and the first vaccines in the U.S. were administered on December 14, 2020 [3]. Vaccination in the TFAS began on December 15 in Puerto Rico, followed by USVI on December 16, Guam on December 17, CNMI on December 19, American Samoa on December 20, RMI on December 29, FSM on December 31, and Palau on January 3, 2021 [personal communications]. The Moderna vaccine received EUA on December 18, 2020, and the Janssen/Johnson & Johnson vaccine received EUA on February 27, 2021. Early in the distribution process, U.S. states received weekly allotments of vaccine, while most of the TFAS received monthly allocations of vaccine doses, inclusive of first and second doses, mainly due to geographic remoteness and small population sizes.

The U.S. COVID-19 vaccination program was initially implemented by all jurisdictions in phases due to limited vaccine supply. Jurisdictions tailored the populations eligible to receive vaccine in each phase based on morbidity and mortality risk, local outbreak epidemiology, and population dynamics. In general, populations in the TFAS eligible to receive vaccine in the first three months included people ages 65 and older, people ages 45 and older at high risk for severe COVID-19 outcomes, first responders, essential workers, and adults living in long-term care facilities. For example, Puerto Rico implemented two administrative orders in February and March of 2021 to prioritize vaccination of people ages 60 and older and those with chronic conditions between the ages of 50–59. The timeline of progression through the phases varied by jurisdiction and eligible groups expanded over time, such that by April 2021 all adults across the U.S. and TFAS were eligible to receive vaccines.

### 2.2. Vaccine distribution strategies in the Pacific TFAS

In the first months of the vaccine rollout, the Pacific TFAS rapidly distributed COVID-19 vaccines according to a phased approach based on work-related exposure to SARS-CoV-2 (e.g., healthcare workers) and/or COVID-19-associated morbidity and mortality (e.g., persons with immunocompromising conditions). All the Pacific TFAS used a point-of-dispensing (POD) vaccine distribution strategy for maximum outreach. At these large, centralized vaccination sites, public health and clinical workers were present to provide vaccination services and education to the community. RMI primarily administered vaccines through small, decentralized PODs, mobile PODs, and door-to-door activities. CNMI, Guam, and FSM also augmented community outreach through add-on mobile and temporary PODs. Strategic delivery to outer island populations, where applicable, was incorporated into vaccine planning to ensure equitable access to the entire population. While there were some local variations in timing and inclusion criteria, the following generally represents the early phases of the Pacific TFAS implementation plan:

- Phase 1a (December 14 – 27, 2020) — healthcare workers and long-term care facility residents
- Phase 1b (December 28, 2020 – January 10, 2021) — ages 75 years and older, first responders, and frontline essential workers
- Phase 1c (January 11 – 31, 2021) — ages 65 years and older, 16–64 years with high-risk conditions, and other essential workers
- Phase 2 (February 1 – 28, 2021) — ages 40 years and older
- Phase 3 (March 1, 2021) — ages 18 (Moderna)/16 (Pfizer-BioNTech) years and older

### 2.3. Vaccine distribution strategies in the Caribbean TFAS

During the summer of 2020, the PR Immunization Program began the process of planning and recruitment of healthcare providers for vaccination against COVID-19. Puerto Rico strategically stood up a network of healthcare providers per proposed vaccination phases that quickly scaled from 0 to 650+ on-boarded providers, the largest network the immunization program has ever managed. Using lessons learned from previous disaster relief efforts in the jurisdiction, PR implemented a hub-and-spoke vaccine distribution model (designed due to limited storage capacity) for the implementation of the phased plan. The hub-and-spoke model is a flexible and adaptable solution to distribution of ultracold vaccines in low- and middle-income jurisdictions, particularly those with remote populations. The model was also particularly critical for PR considering consistent electricity needed to maintain the cold-chain was still not available in some remote areas due to the lasting impacts of Hurricane Maria, even in late 2020.

The hub-and-spoke distribution model was designed with one hub in the northern part of the island and one in the south. From the hubs, portable freezers could be transported to “spokes” – clinics and other vaccination sites around the island – allowing flexibility in the distribution of vaccines to the population while ensuring cold-chain maintenance throughout the distribution process using portable back-up generators. The hub-and-spoke model enhanced the accessibility of the COVID-19 vaccines to the 78 municipalities in PR and helped to overcome the challenges related to ultracold storage requirements. This model allowed distribution of COVID-19 vaccines to a large network of traditional and non-traditional healthcare professionals that included hospitals, doctor’s offices, pharmacies, specialty clinics, community-based organizations, federally qualified health centers, laboratories, and the private sector.

The USVI also utilized a hub-and-spoke model, with centralized storage and distribution on the islands of St. Croix and St. Thomas, based on availability of deep freeze storage capacity. This process required USVI to build a new distribution strategy due to the cold storage availability.

The hub-and-spoke model in the Caribbean territories allowed effective distribution while preserving the integrity of the vaccine and reducing vaccine waste. This model allowed flexible network expansion or reduction based on situational and local needs. The model also allowed on-time delivery of vaccines and compliance and cadence with first and second doses and subsequent booster doses. As a result, PR – with a poverty rate of approximately 43% and a healthcare system left fragile after recent natural disasters – was able to successfully overcome barriers to become one of the leading jurisdictions in vaccination coverage by October 2021 [14].

### 2.4. Collaborations

Globally recognized success in mitigating COVID-19 in the TFAS was driven by consistent and high-performing strategic partnerships with internal and external stakeholders. While some of these relationships existed prior to the pandemic, jurisdictions also forged partnerships and collaborations with new stakeholders to expand their capacity to respond to COVID-19. Jurisdictions and their partners built upon strategies and lessons learned from prior vaccine-preventable disease outbreaks and natural disasters; however, the complexity of the COVID-19 pandemic required a new level of multi-partner coordination and collaboration across the TFAS. These multilateral and inter-jurisdictional partnership activities enabled region-wide coordination, partner harmonization, and the enhancement of response strategies to address the isolated and especially under-resourced jurisdictions of the TFAS. Building collaboration across U.S. government agencies and support partners enabled a successful COVID-19 response in one of the populations at highest risk of severe outcomes for COVID-19 in the U.S.

For example, key partnerships between USVI Department of Health (DOH) and various local and federal government agencies were critical to the success of the vaccination rollout. Collaborations with the Office of the Governor, Office of Management and Budget, and Department of Finance enabled the immunization program to provide incentives for vaccinations by significant dates and for certain populations. Another important collaboration in USVI was the partnership with the National Guard, which included support with U.S. Army mission assignments, staffing of the Community Vaccination Centers, and providing logistical support such as vaccine transportation to improve vaccine access for underserved populations.

CDC subject matter experts (SMEs) assisted jurisdictions across many facets of the vaccination program, including distribution logistics, monitoring and evaluation, risk communication, and vaccine confidence evaluation and improvement strategies. For example, in one state of FSM, what appeared to be low vaccine uptake was assumed by local leaders to be a result of vaccine hesitancy in the jurisdiction. In response, an Epi-Aid team was deployed to investigate and develop strategies to increase vaccine uptake. In the end, the team determined vaccination coverage estimates were artificially low due to inflated denominator estimates. CDC SMEs uncovered a similar finding in USVI, where COVID-19 vaccination coverage using the 2020 Decennial Census population count (a data source not available during initial vaccine implementation) was 12 percentage points higher than coverage using the 2010 Decennial Census count, when comparing on an absolute scale (63% vs. 51%). These findings underscored the importance of collaborations between local health departments and supporting government agencies to identify and mitigate data quality concerns in monitoring the vaccine rollout.

While data quality was determined to be the main driver of apparent low vaccination coverage in the FSM state, the Epi-Aid team did determine there was at least some level of vaccine hesitancy in the population as was initially suspected. According to key informant interviews among FSM immunization program staff and clinicians conducted by CDC in 2014 related to attitudes and barriers to vaccination, vaccine hesitancy was not indicated to be a major concern or barrier to routine vaccination (data not published). Recent studies since the pandemic have also indicated vaccine hesitancy in the Pacific region and the Americas are similar, and among the lowest globally [15]. However, misinformation about the COVID-19 vaccine was reported across most TFAS jurisdictions as a driver for hesitancy toward the new vaccine [16]. To address this concern, the team in FSM coordinated with state and national health officials to launch a coordinated campaign aimed at addressing misinformation and educating the community on the importance of vaccination. The team acknowledged collaboration with local community organizations and faith-based leadership, a model adapted from previous vaccination campaigns, as a key to their success [17].

In the USAPI, engaging faith-based leaders has been a strategy used during past vaccination campaigns, and notably during the COVID-19 response, as a vital means of disseminating information, addressing community concerns, and promoting vaccination campaigns. Immunization programs in the region were able to appeal to faith leaders through demonstrating the potential vaccination has to protect lives and promote equity. Similarly in PR, where studies indicated an association between greater religiosity and lower perceived susceptibility toward COVID-19 disease and less willingness to get the COVID-19 vaccine, the PR DOH partnered with local faith leaders to locate test-to-treat centers at their churches [18]. The PR DOH cited using trusted community messengers alongside community health extension workers from the area DOH who attend their local church as key facilitators in garnering support from the faith leaders to collaborate on the response.

Additional strategies to address vaccine hesitancy were implemented in the Caribbean territories. In the USVI, CDC SMEs collaborated with DOH to establish a series of radio and newspaper interviews to address vaccine hesitancy in the population. Approximately 30 radio interviews and 5 newspaper interviews were completed by SMEs working in the region. A vaccine educational campaign was developed in the USVI to provide facts and dispel myths at the weekly Government House Press Briefings; the immunization program also conducted small group informational sessions with local organizations providing personal interactions with infectious disease physicians to answer concerns about the vaccines among providers. Another creative strategy to increase vaccine confidence in the USVI involved partnering with a local musical artist to have an on-stage vaccination during a live concert event.

**Fig. 1.**
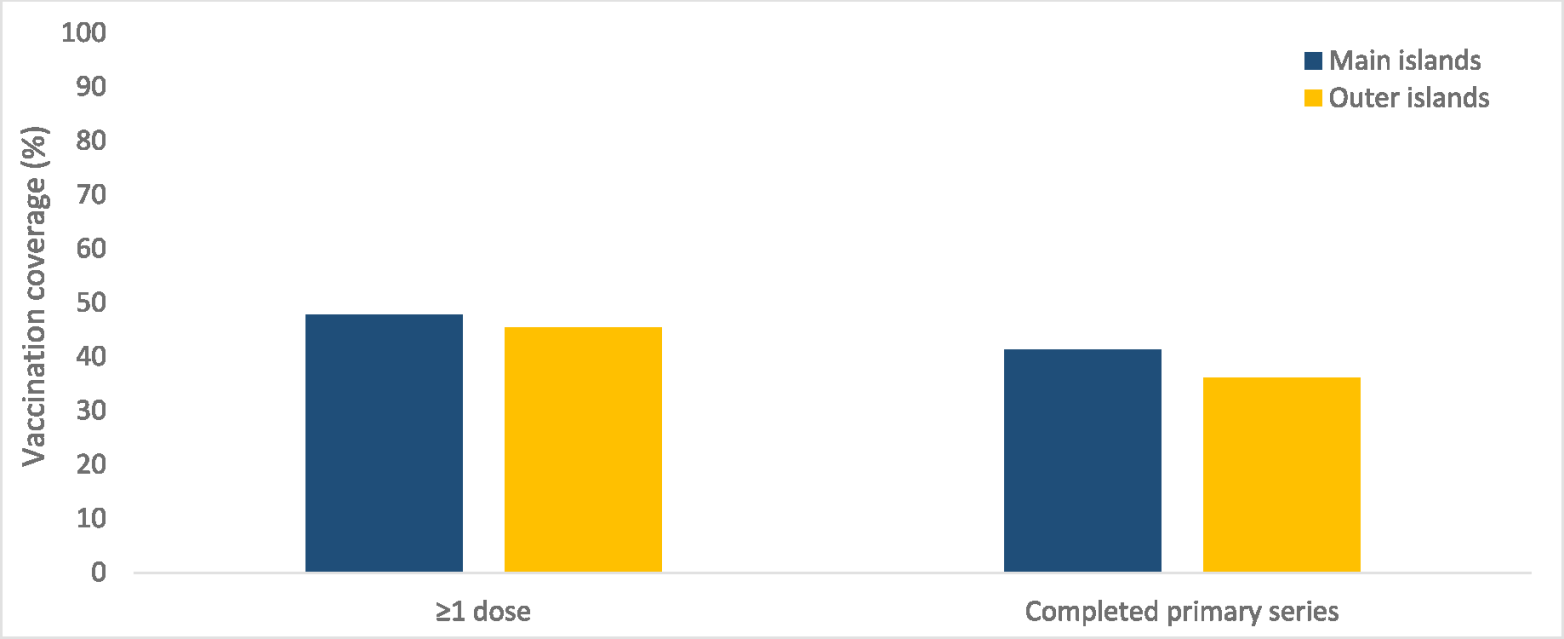
COVID-19 vaccination coverage* among adults ≥18 years as of June 15, 2021, by region – Federated States of Micronesia. *Completed primary series defined as receipt of one Janssen/Johnson & Johnson vaccine or two doses of original, monovalent mRNA vaccines. Data from jurisdictional immunization information system.

Immunization programs in the TFAS also had a need to rapidly assess vaccine uptake among populations recommended for vaccination. A weekly small-area vaccination coverage report was developed in early 2021 by CDC vaccination assessment SMEs in collaboration with Pacific TFAS immunization program managers. The report provided detailed information at village or outer island level on vaccination coverage and geographic gaps in vaccine uptake. The USAPI immunization programs utilized this powerful data for action, strategically providing vaccine access to historically under-served outer island populations. As a result of innovative vaccine distribution strategies coupled with new data monitoring tools, COVID-19 vaccination coverage among populations living in the outer islands of Pacific TFAS was similar to vaccination coverage among populations living on the main islands by June 2021, representing a substantial reduction in disparities compared to historical routine vaccination coverage (Fig. 1).

Strong communication among response partners and healthcare providers was also critical to the success of the vaccine rollout. CDC and immunization programs collaborated to implement new communication strategies, such as weekly office hours with providers. These weekly virtual meetings shared new CDC guidelines, local policy changes, best vaccine management practices, trainings, and culturally appropriate messages to increase vaccine confidence. In PR, over 56 teleconferences with providers were conducted during the first year, and more than 15,000 health professionals benefited from those discussions; mandatory training was conducted with over 25,000 vaccinators. An evaluation conducted in early 2021 found strong adherence to best practices and no major deficiencies that could jeopardize vaccine viability or patient safety among a sample of vaccine providers across all 7 health regions, indicative of effective communications and training strategies [19].

CDC’s COVID-19 Response team, including Health Department Liaisons, CDC field staff, and Office of Island Affairs, worked closely with Pacific jurisdictional health officials, Administration for Strategic Preparedness and Response (ASPR), Federal Emergency Management Agency (FEMA) officials, and other support partners in the Pacific, such as the World Health Organization (WHO) and Pacific Island Health Officers’ Association (PIHOA). Weekly coordination calls with all partners established firm lines of communication and enabled strategic planning between entities well before vaccine arrived in the region and continued throughout the initial rollout phase.

Private sector partnerships were also an innovative strategy for some TFAS jurisdictions. The USVI immunization program allocated a high proportion of vaccine to high capacity, high throughput private healthcare partners and private medical groups, and gave private providers access to the CDC Vaccine Administration Management System (VAMS) to be able to alleviate reporting pressure on the program. Some private sector providers set up mass vaccination sites for ease of access – to supplement the USVI government-run sites. Mobile vaccination sites on various days in high traffic areas and on-demand events hosted by long-standing community clinics were new approaches that helped expand access to vaccines across the jurisdiction.

### 2.5. Vaccination program outcomes

The TFAS, which initially implemented strict border controls to protect their health systems and populations, rapidly distributed COVID-19 vaccines to protect their populations and facilitate reopening of trade and travel.

During the initial vaccine rollout, doses were distributed to jurisdictions participating in the U.S. domestic vaccination program pro rata based on population. Within the first month of the program, more than 416,950 vaccine doses had been distributed to the TFAS and at least 187,394 adults received their first vaccine dose (Supplementary Table 3; vaccine distribution data not available for Palau).

Palau was the first TFAS to reach >80% of their adult population (ages 18 years and older) vaccinated with at least one dose in May 2021, followed by American Samoa, CNMI, Guam, and RMI in July 2021. The U.S. overall did not reach >80% adults with at least one dose until November 2021 (Supplementary Table 3).

**Fig. 2.**
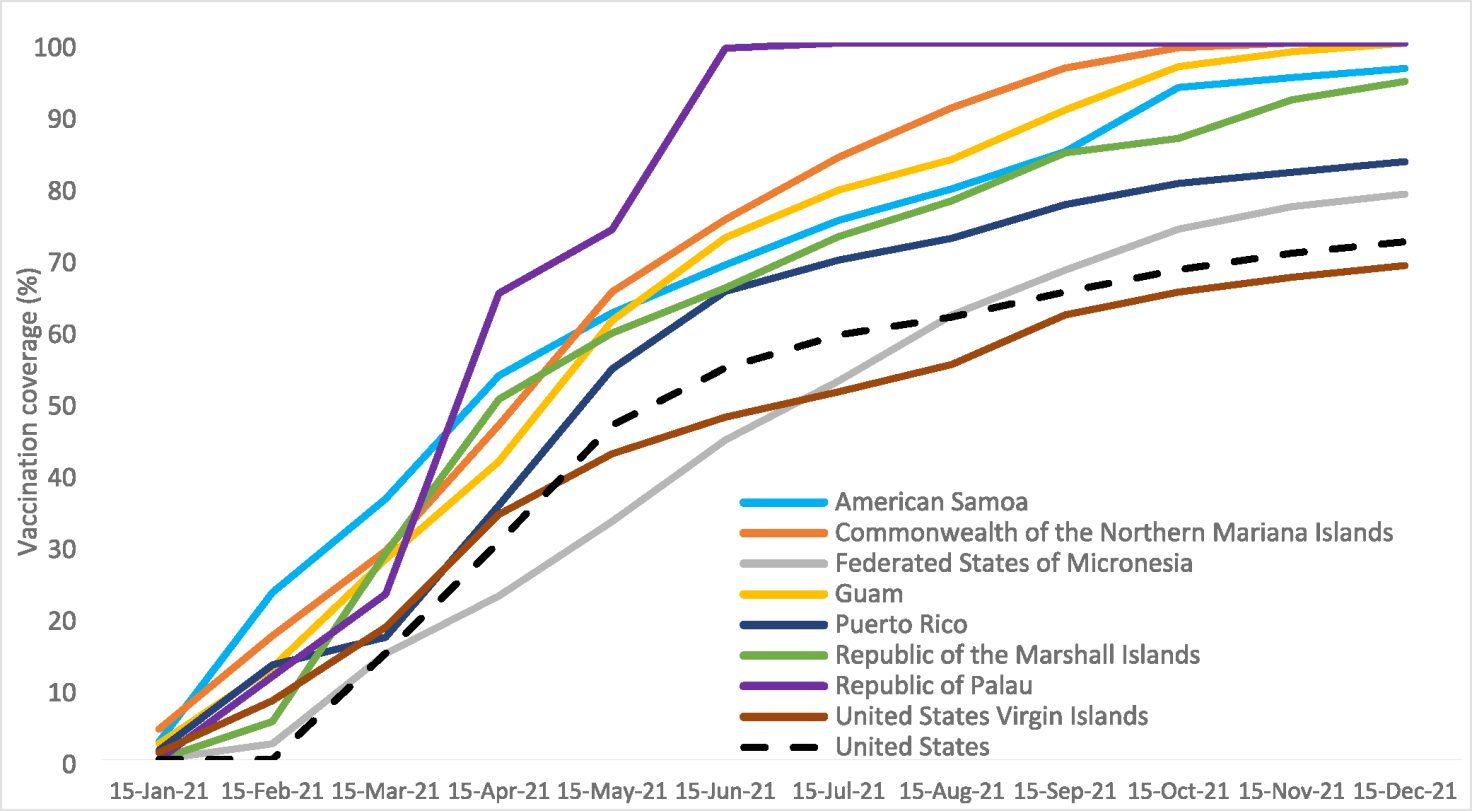
Completion of COVID-19 primary series* among adults ≥18 years, by jurisdiction – 2021. Abbreviations. CDC = Centers for Disease Control and Prevention; CNMI = Commonwealth of the Northern Mariana Islands; FSM = Federated States of Micronesia; PR = Puerto Rico; RMI = Republic of the Marshall Islands; USVI = United States Virgin Islands; US = United States. *Completed primary series defined as receipt of one Janssen/Johnson & Johnson vaccine or two doses of original, monovalent mRNA vaccines. Vaccination coverage data for American Samoa, CNMI, FSM, Palau, and RMI from jurisdictional immunization information systems. Vaccination coverage data for PR and USVI from internal data provided by respective immunization programs. Vaccination coverage data for US from CDC COVID Data Tracker, accessed at https://data.cdc.gov/Vaccinations/COVID-19-Vaccinations-in-the-United-States-Jurisdi/unsk-b7fc.

During the first six months of the vaccination program, completion of the primary series among adults 18 years and older in American Samoa, CNMI, Guam, Palau, Puerto Rico, and RMI outpaced the U.S. overall each month (Supplementary Table 3, Fig. 2). Within eight months of the vaccine rollout, completion of the primary series was at or above 80% among adults in four of the TFAS, and all TFAS jurisdictions except USVI reached >80% completion of the primary series before the end of the year (Supplementary Table 3, Fig. 2). According to National Immunization Survey data, the U.S. overall reached 80% completion of the primary series among adults in January 2022 [20].

## 3. Conclusion

The first year of the COVID-19 pandemic posed a difficult challenge for U.S. public health systems, particularly in the TFAS. However, the TFAS were successful in limiting the impact of COVID-19 through thoughtful planning, implementation of novel approaches to vaccine delivery and vaccine confidence (tailored to specific jurisdictional considerations), and strong collaborations across U.S. government, local, and regional partners.

Lessons learned from the TFAS COVID-19 vaccine rollout, particularly use of innovative strategies to increase vaccine access, monitor uptake, and reduce disparities can be applied to routine vaccination services to improve uptake and reduce the spread of vaccine-preventable diseases in the TFAS and other resource-constrained island nations.

As an example, in the USAPI the newly strengthened collaborations and novel tools developed to monitor COVID-19 vaccination coverage are now being leveraged to improve performance across their vaccination programs. In these jurisdictions, CDC assessment SMEs are working on enhancing existing routine vaccination coverage products to include local-level data, similar to the COVID-19 vaccine reporting strategies, which will better inform decision-making and use of limited resources in reaching remote populations. These improved data products are currently being shared with immunization programs and their supporting external partners, such as WHO, to develop program performance improvement plans across all routine vaccines [internal communications]. These tools were proven effective in planning vaccine distribution strategies that were aimed at decreasing geographic disparities for COVID-19 vaccine and may be an effective tool for reducing disparities for other routine vaccines as well.

The leveraging of governmental and non-governmental partnerships and the tailored approaches to improving access to vaccination services equitably will be key to strengthening immunization program performance for all routine vaccines in the TFAS and similar settings moving forward.

## Supplementary Material

Supplementary Tables

## Data Availability

Data will be made available on request.
